# New Horizons in Dermal and Transdermal Drug Delivery Systems

**DOI:** 10.3390/ph16121654

**Published:** 2023-11-28

**Authors:** Joana Marto, Sandra Simões

**Affiliations:** Research Institute for Medicines (iMed.ULisboa), Faculty of Pharmacy, Universidade de Lisboa, Avenida Professor Gama Pinto, 1649-003 Lisbon, Portugal

Dermal and transdermal drug delivery represents an important strategy to target drugs towards the site of action or to noninvasively enhance treatment activity, circumventing the hepatic first passage and reducing toxicity [1]. However, dermal and transdermal delivery faces important issues, such as the stratum corneum barrier effect, the delivery of the drug to the skin tissue, and the passage through the skin complex structure to reach the lymphatic and vascular compartments [2,3]. Additionally, the low amount of a drug that can be delivered through the skin can compromise the drug’s therapeutic effect. To solve such issues, the development of advanced systems based on passive or active approaches has enlarged the number of drug candidates that can be delivered through the skin [4,5,6]. Especially, nanotechnological approaches have all opened new horizons in this field and emerged as attractive administration methods [7,8]. This is, thus, an exciting era for skin drug delivery development and presents enormous opportunities considering that it configures a noninvasive administration approach with increased patient compliance. We hope to demonstrate the potential of drug delivery systems specially conceived for to-the-skin and through-the-skin delivery when the skin is the target organ and when the skin the route of entry of substances into the body.

We have great pleasure in accepting the invitation to be guest editors for this Special Issue of “*Pharmaceuticals*”. This volume presents review and original research papers by experts on areas relevant to the topic of “New Horizons in Dermal and Transdermal Drug Delivery Systems” and includes contributions relevant to pharmacists, dermatologists, and other researchers working on the special topic of dermal and transdermal drug delivery. The contributions are listed below.

It is generally recognized that the evaluation of the oxidative state of the skin plays a key role in further understanding and prevention skin disorders [9]. The first contribution presents a novel ex vivo model to evaluate the skin oxidative state of the stratum corneum by the measurement of its antioxidant capacity (AOC) combining ORAC assay with tape stripping and infrared densitometry. This combination allows the evaluation of the intrinsic AOC (iAOC) and variations in the iAOC become distinguishable when caused by various skin treatments, i.e., several factors were found to impact the iAOC being possible to elaborate guidelines for future research using this method.

Microemulsions (MEs) are isotropic thermodynamically stable lipid-based pharmaceutical systems with a high potential to increase drug skin permeation of drugs through the skin [10,11]. The second contribution developed imiquimod (IMQ)-loaded MEs based on phospholipids and oleic acid to enhance IMQ penetration into the epidermis. Results on ex vivo skin deposition assay demonstrated the ability of 1% IMQ MEs to deliver to the epidermis similar IMQ amounts as the commercial product containing five times more IMQ and suggesting a lower risk of systemic absorption compared to the commercial product. The third contribution also developed MEs as carriers to improve the solubility, permeability, skin whitening, and bioavailability of genistein. The results showed that formulation factors, such as oil type, HLB value, and surfactants and cosurfactants combinations had a significant impact on the solubility and permeability of genistein. The pharmacokinetic study indicated the relatively higher bioavailability of genistein transdermal administration compared to oral administration. Genistein transport across the skin was confirmed by the whitening effect and the good compatibility of MEs with the skin was achieved.

Nanosystems have been used with success in several areas of skin disease management, and the special case of nanofibers is emerging as a nanotechnology-based system with excellent wound-healing properties [12]. This was confirmed by the fourth contribution study, which developed silk sericin-based hybrid nanofibrous mats loaded with ferulic acid for diabetic foot ulcers, prepared via electrospinning. In vitro release demonstrated a sustained FA release from nanofibers over an extended period. Moreover, the excellent wound-healing efficiency of the nanofibers was achieved in vivo in diabetic rats. Hesperidin, a bioflavonoid extracted from various citrus fruits, described as having wound-healing properties, has low solubility and would benefit from a technological strategy to improve its therapeutic efficacy, namely, to enhance the delivery of hesperidin to endogenous sites in the wound bed. The research in the fifth contribution is based on the development and characterization of a novel system for the incorporation of hesperidin, namely, lipid-polymer hybrid nanoparticles. The study suggested that the interaction between the ratio of surfactants and lipids affects particle size or decreases particle size as a function of drug concentration. Through DSC and XRD, it was possible to determine the physical stability of the loaded drug in the hybrid system. In vitro drug studies confirmed hesperidin’s sustained release, an important achievement considering the bioactive deliver to wounds.

The sixth contribution is a literature review on the cannabis- and cannabinoid-based products for the treatment of skin inflammatory diseases, starting with the role of the cutaneous endocannabinoid system in several skin inflammatory diseases and ending with the legislation on cannabis use in medicine and currently approved therapeutics. The paper suggests that cannabis and cannabinoid receptor modulators have therapeutic actions in several inflammatory skin diseases [13,14,15,16,17,18] due to their anti-proliferative, immunomodulatory and anti-inflammatory actions. Even considering that cannabinoid-based medicines to treat skin disorders are not yet available on the market, preliminary evidence of the potential benefits have been reviewed. Despite the successful investigation into cannabinoid-based (nano)formulations [19], available clinical trials are scarce and do not provide enough safety and efficacy.

In summary, this Special Issue of *Pharmaceuticals* highlights the challenges of dermal and transdermal drug delivery, with a special emphasis on skin disease-oriented developments. Wounds still present a threat to public health and this issue brings new advances on carrier-mediated enhancement of drug activity to wound management. This Special Issue also provides methodological updates related to the evaluation of oxidative stress in skin and new therapeutic perspectives on treating skin diseases, namely, the possible therapeutic potential of cannabis, essentially the pharmacologically active cannabinoid compounds, for the treatment of skin inflammatory diseases. In this Special Issue of *Pharmaceuticals* dedicated to dermal and transdermal delivery, we present a selection of research papers mainly focusing nanoparticulate systems.
